# Energy, Transportation, Air Quality, Climate Change, Health Nexus: Sustainable Energy is Good for Our Health

**DOI:** 10.3934/publichealth.2017.1.47

**Published:** 2017-02-16

**Authors:** Larry E. Erickson, Merrisa Jennings

**Affiliations:** 1 Department of Chemical Engineering, Kansas State University, Manhattan, KS 66506; 2 Department of Biological and Agricultural Engineering, University of Arkansas, Fayetteville, AR 72701

**Keywords:** sustainability, solar, electric vehicles, shade, infrastructure, parking lots, economics, air quality, renewable energy

## Abstract

The Paris Agreement on Climate Change has the potential to improve air quality and human health by encouraging the electrification of transportation and a transition from coal to sustainable energy. There will be human health benefits from reducing combustion emissions in all parts of the world. Solar powered charging infrastructure for electric vehicles adds renewable energy to generate electricity, shaded parking, and a needed charging infrastructure for electric vehicles that will reduce range anxiety. The costs of wind power, solar panels, and batteries are falling because of technological progress, magnitude of commercial activity, production experience, and competition associated with new trillion dollar markets. These energy and transportation transitions can have a very positive impact on health. The energy, transportation, air quality, climate change, health nexus may benefit from additional progress in developing solar powered charging infrastructure.

## 1. Introduction

The Paris Agreement on Climate Change of December 2015 [1] has the potential to benefit all people. At this conference, the United Nations prioritized several goals, including reducing carbon emissions, which are critical to maintaining healthy populations and ecosystems. If these goals can be accomplished quickly, then the rates of change of the anthropogenic effects such as global warming, rising sea levels, and acidification of oceans can be lessened. This will have great value for human health and the environment. The transitions to generate all electricity without carbon emissions and to electrify transportation will take many years and cost trillions of dollars; however, these changes are central to meeting the goals of the Paris Agreement and reducing the impacts of climate change on human health.

The objective of this work is to address the issues at the nexus of energy, transportation, air quality, climate change and health. By reducing carbon emissions, the health issues associated with climate change and air pollution will be impacted positively. There are many authors who have addressed various parts of this nexus [1]–[55]. Electric power generation and transportation combined produce more than 50% of the carbon emissions associated with combustion, but this percentage may decrease rapidly with the transition to wind and solar power generation and electric vehicles.

To make transportation truly combustion free in order to decrease air pollution and improve human health, vehicles will need to transition to electric power fueled by emission free renewable energy. Solar powered charging infrastructure for electric vehicles (EVs) is one of the transitions that is at the heart of this nexus [2]. One way to advance this transition is by covering parking lots with solar panels and adding EV charging infrastructure (also known as EVSE—electric vehicle supply equipment). One added benefit of initiating this method is that the shade provided by the solar panels will have positive effects by decreasing heat related health issues and property damage.

Academic research and industrial development on solar panels, batteries, and electric vehicles have contributed to progress toward the transition to sustainable energy [2],[22]. Additional research and development associated with the smart grid, which is important for the transition to wind and solar generated electricity, are needed in order to transition to real time prices for electricity. Electric vehicles have the potential to contribute positively to grid stability when real time prices for electricity are made available and optimized [2].

Greenhouse gas emissions and air pollutants are directly connected because carbon dioxide and air pollutants such as PM_2.5_ and nitrogen oxides are produced by combustion of motor fuels, coal, and other substances.

## 2. Human Health Issues

Along with the environmental degradation, there are many human health issues associated with climate change, energy, transportation, and air quality [3]. From the coal mines to coal burning power plants, there are environmental health impacts. Many are associated with air quality. Along with the immediate effects of air pollution, the long term effects of increasing temperatures will also be quite problematic. Since the acceleration of global warming is happening too quickly for the human species to evolve, heat related health problems will result in deaths and illness due to heat strokes, hyperthermia and exhaustion [3].

Air quality is impacted by pollution associated with combustion processes that includes smog, soot, acid rain, and toxic air emissions [27]. There are about 6.5 million deaths each year due to air pollution according to the World Health Organization (WHO) [4]. Poor urban air quality associated with coal burning power plants and transportation has caused health problems for many people and some deaths. Reductions in carbon emissions are generally beneficial to air quality and health. Climate change is expected to impact asthma and allergies by extending the growing seasons of vegetation that emit pollen [28]. Global warming will also lead to more wildfires and dust storms which emit particulates that impact air quality and health [3].

Along with extreme events such as, wildfires, and dust storms, flooding also impacts health [3]. Along with the immediate dangers to human life, such as transferring more pollutants to water sources and increasing exposure to vector borne diseases, flooding destroys essential infrastructure and agricultural ecosystems, which can lead to long term community development issues. The less a community can divert funds from infrastructure to public health, the less care each individual can afford to receive. In 2016, flooding in southern Louisiana damaged at least 60,000 homes and led to 13 deaths [38]. Increased global temperatures have likely contributed to a number of unprecedented recent floods [38]. In Louisiana the Amite river crest was 1.5 m above the 1983 record [38].

Climate change alters agricultural environments, food supplies and product quality. As mentioned previously, climate change will lead to more severe droughts, and long periods without rainfall impact farming negatively. Although water can be transported to the crops, this process is extremely energy intensive and may not be worth the rate of return. Due to these facts, food supply and food security are expected to be impacted negatively by climate change [3].

The events associated with climate change are expected to not only affect physical health but also affect mental health by disturbing quality of life for many individuals. There will be those who need to move and those who choose to move because of the impacts of climate change. Governments and communities will have events associated with climate change that make it difficult or impossible to properly care for the physical and mental health needs of their people [5].

Black et al. [40] point out that the availability and reliability of ecosystem services and the exposure to hazards such as flooding may contribute to decisions to move to a new location. The Intergovernmental Panel on Climate Change expects significant population movements because of climate change [40],[41]. All people want to have an environment where they feel safe and secure, and this desire contributes to human migration.

The following is an example associated with climate change from July 2016. Thirteen people were hospitalized and about 1500 reindeer died from an outbreak of anthrax in western Siberia. The summer thawing was greater than normal and this outbreak has been attributed to an infected reindeer carcass that had been frozen and covered with snow and ice [6]. Revich and Podolnya [7] have pointed out that risks associated with zoonoses are expected to increase because of climate change. This health risk due to climate change is a present risk and a future risk.

Satish et al. [15] have investigated the effect of carbon dioxide concentration on human decision-making performance. Concentrations of 600, 1000, and 2,500 ppm were used in a work performance environment, and the results showed that performance decreased significantly at the higher carbon dioxide concentrations. Thus, it is expected that the increases in carbon dioxide concentrations with time due to carbon emissions will have a health impact associated with them. The value in the atmosphere is presently about 400 ppm in 2016 and rising. In buildings with human occupants, values of about 600 ppm have been reported [15].

## 3. Energy and Air Quality

Combustion of fossil fuels causes short term threats such as air pollution and long term threats like climate change through the emission of carbon dioxide. Coal fired power plants have been an environmental concern because they are a significant source of carbon emissions and health damaging air particulates. Despite the immediate effects they have on the health of surrounding communities, some coal burning power plants are kept in service for more than 50 years. One alternative to coal is natural gas. Natural gas is a cleaner fuel that is used in many power plants that are operated to generate electricity to meet peak power requirements. It emits less carbon dioxide per unit of energy produced as compared to coal. Due to the immediate threats of coal combustion, one of the higher priorities is to reduce the use of coal for heating, power generation, and industrial processes.

## 4. Transportation, Air Quality, and Health Nexus

Transportation has a negative impact on health because of its negative impact on air quality. The particulates in diesel exhaust often contain organic compounds that are associated with cancer [2],[8],[9]. Vehicle emissions, including particulates, nitrogen oxides, and volatile organic compounds, are elevated in urban areas, and they impact human health [2]. The global health impacts associated with air pollution have economic costs of more than one trillion dollars per year [2],[13],[14] and there are many deaths attributed to the impacts of transportation on air quality.

Particulate matter and black carbon in particulate matter have significant impacts on human health with an estimated 2.9 million attributable deaths in the year 2013 [46]. Ambient particulate matter in air is the sixth largest overall risk factor for global premature mortality [47]. It is a major concern in most cities with population of 5 million or more residents [43].

The particulate matter in air associated with combustion includes emissions from transportation, coal fired electricity generation, industrial processes and cooking of food with solid fuels [42]–[45]. The World Health Organization Air Quality Guideline for PM_2.5_ is 10 micrograms/cubic meter in air [46]. This is an annual mean value. In the United States, the regulatory value is 12 and in Europe it is 25. Approximately 98% of cities with populations over 100,000 do not meet the WHO guideline in low and middle income countries [42]. Bauer et al. [46] report population-weighted annual average values of PM_2.5_ for 2013 for several countries: China-54.3, India-46.7, Nigeria-29.5, and United States-10.7. In major cities reported values are as follows: Delhi-170, Dhaka-135, Cairo-66, Lagos-62, Beijing-55, Lima-39, Moscow-22, Los Angeles-19, London-15, and Chicago-13 for PM_2.5_ in micrograms/cubic meter [43].

In London, UK, nitrogen oxide emissions are of significant concern because the regulatory limit value of 40 micrograms/cubic meter is not being met [48]. Efforts to reduce nitrogen oxide emissions in central London include support for zero emission taxis, safe cycling measures, and a policy of no unnecessary idling [48]. It is estimated that more than 4000 people have their life cut short each year due to poor air quality in London [48].

There are many efforts to improve urban air quality [1]–[3],[14],[18],[27],[36],[48]–[50]. In India health benefits are included in transportation planning [49]. In Norway there has been a significant effort to encourage the purchase and use of plug-in electric vehicles [50],[51]. Because of incentives, Norway has reached the point where more than 25% of new car sales are plug-in electric vehicles [51]. Some of these are plug-in hybrid automobiles. Urban air quality in Norway is being improved because of progress in the electrification of transportation and the generation of electricity with renewable energy [2],[50].

The estimated benefits of reducing emissions to achieve a global concentration of 12 micrograms/cubic meter in all cities would result in 46% reduction in global mortality associated with particulates with reductions of 4% in the United States, 20% in the European Union, 69% in China, 49% in India, and 36% in Pakistan [55]. This is a reduction of more than 1 million/year [55].

California has been making significant progress in its effort to improve air quality by encouraging the electrification of transportation through incentives [2]. There are health benefits associated with the reduction in concentrations of air pollutants [36].

## 5. Renewable Energy, Electric Vehicles, Energy Storage, Charging Infrastructure, and Health Nexus

The transition to sustainable energy and sustainable transportation has the following major challenges:

1. We need to generate all electricity without carbon emissions. Wind and solar energy can be expanded to generate more electricity.
2. We need to electrify transportation and travel without generating carbon emissions. There are many new challenges to address to electrify all ground transportation.
3. We need to improve air quality by reducing pollution associated with carbon emissions. Air quality will be much better if we accomplish 5.1 and 5.2.
4. We need to enhance charging infrastructure for electric vehicles. One way to accomplish this is with solar powered charging stations [2],[10]–[12],[37]. There are many dimensions associated with this challenge. Workplace charging, fast charging along highways, and charging at other locations are all important. The concept of changing batteries quickly at roadside stations is also a viable alternative.
5. We need to expand the infrastructure for safe bicycle transportation. In some places in Europe there are excellent pathways for bicycles. This needs to be extended to other parts of the world.
6. We need to improve and electrify public transportation. There are many places in the world where air quality and health would be better if public transportation was powered by electricity rather than diesel fuel. Electric buses are available to replace diesel powered buses.
7. We need to modernize the electrical grid. The modernization of the electrical grid with better communication and information is very important to accomplishing some of the goals above.
  There is a need to have real time prices or time of use prices for electricity because wind energy and solar energy need to be used when they are generated or stored for future use. There are several electrical loads that can be shifted in time (washing and drying clothes and charging electric vehicles are examples). A modern electrical grid with good communication features that will allow the effective implementation of time of use prices can be very helpful in efforts to manage an electrical grid with large percentages of wind and solar power generation.
8. We need to use electric vehicles to store electricity. The growth in numbers of electric vehicles and the growth in the capacity of EV batteries have the potential to be very helpful to the management of electrical power generation and distribution. If every family had an EV with 50 kWh of battery storage, this would provide storage for more than the present daily use of electricity by a typical family. Daily home use may be about 10 to 20 kWh while transportation use may be 10 to 15 kWh/day (30 to 45 miles/day or 48 to 72 km/day). With many electric vehicles in service, about half the electricity flow from the grid can be shifted in time to off peak times when it is easier and less expensive to supply it.
9. We need to continue innovative research to develop new technologies and approaches [2],[52].
10. We need to continue to advance public policies and cooperative efforts to introduce new innovative technologies that have benefits to society.
11. We need to have a global technology transfer program so new ideas and developments are communicated to all who look for advances on the Internet.

## 6. Economic Aspects

The impact of climate change on economic production has been estimated to be about 23% by Burke et al. [16]. If no action is taken to address climate change and reduce carbon emissions, in 2100 economic production will be 23% lower than if there were no climate change. In another study, the annual cost of climate change in the U.S. is estimated to be about $270 billion/year in 2025 [19]. The gross domestic product in the U.S. was about $18 trillion in 2015 and the gross world product was about $107 trillion in 2014.

Health care costs are a significant portion of national and global expenditures. In the United States, about $3 trillion is spent on health care annually. The global expenditures for health are more than $6 trillion/year [17]. There is the potential to reduce health care costs by reducing carbon emissions and improving air quality at the same time. The potential value of air quality improvements in terms of reduced health care costs and better health in the U.S. are in the range of $37–90 billion/year [2]. The American Lung Association in California report [36] estimates that the harm in 2015 associated with air pollution attributed to passenger vehicles in the ten states of California, Connecticut, Maine, Maryland, Massachusetts, New Jersey, New York, Rhode Island, and Vermont was $24 billion for health and $13 billion for climate. In China, health costs associated with air pollution are about 1.2–3.3% of China's gross domestic product [2],[18]. The social costs of the health impact of outdoor air pollution were about $1.3 trillion/year in 2010 in China and about $0.6 trillion/year in India. This can be compared to $1.7 trillion/year for the 35 OECD countries [53]. The estimated cost of serious health consequences because of particulate matter pollution is about 3% of India’s GDP; 1.7% is from outdoor and 1.3% is from indoor air pollution [54]. In India, there are significant health and economic benefits associated with taking action to reduce particulate matter in urban air [53],[54].

Each economic study has assumptions and considerations with respect to what is included in the study. These have some impacts on the results that are presented. The important message in this work is that there are very significant social, environmental, and economic costs associated with particulate matter in air in large cities and metropolitan areas in many parts of the world.

The International Energy Agency report indicates that air quality and health can be improved very significantly by spending 7% more on energy [4].

In the U.S. more than $1 trillion is expended each year for purchases and investments related to transportation. The average annual expenditure per household for transportation is $8,000 or about 17% of annual spending [20]. Transportation is about 8.7% of gross domestic product [20]. Housing, health care, and transportation are the three largest parts of most family budgets.

The global expenditure for electric power generation, distribution and use is more than $1 trillion/year. One of the most important transitions associated with reducing carbon emissions and improving health is the transition from coal burning power plants to wind and solar production of electricity. Because of advances in technology, wind energy and solar energy are becoming more competitive, and prices of wind and solar power generation have trended downward for the last several years. In 2015 in the U.S. for new power generation capacity, wind was first and solar was second. Lester Brown [21] and coworkers have authored The Great Transition, which describes the shifting from fossil fuels to wind and solar energy.

The prices for electric vehicles are related to the prices for batteries. Battery prices have been decreasing rapidly because of technological developments, production experience, and competition [2],[22]–[26]. The reduction in battery costs is one of the reasons that sales of new electric vehicles have continued to increase. Customers have more and more options to select from also. Global annual sales of EVs were more than 60,000 in June 2016; they will soon reach one million/year.

Trancik and coworkers have investigated the vehicle, fuel and maintenance costs of 125 light duty vehicle models on the U.S. market [39]. They have reported that consumers are not required to pay more for a low-carbon-emitting vehicle.

One of the benefits to society of the efforts to electrify transportation and reduce greenhouse gas emissions is the reduction in the price of gasoline and diesel fuel that has taken place since 2013. There is reduced demand for fuels because of EVs, hybrid vehicles, and efficient gasoline powered vehicles. The federal policy to improve efficiency has already impacted air quality positively in California [2].

## 7. Solar Powered Charging Infrastructure for Electric Vehicles

There is general agreement that it would be beneficial to reduce carbon emissions, improve air quality, and have better health because of the electrification of transportation and the transition to the generation of electricity using sustainable energy [1]–[5],[13]–[19],[21]–[25],[37]. Williams et al. [24] have shown that carbon emissions can be reduced substantially by electrifying transportation and transitioning to renewable sources to generate electricity. A book [2] and a comprehensive review paper [37] have been published recently on solar powered charging in parking lots.

Solar panels can be added to many parking lots to provide shaded parking, and an infrastructure of charging stations can be part of these projects. If 200 million parking spaces are modernized with solar panels and electric vehicle supply equipment (EVSE), about 1/4 of the electricity that is produced today in the USA (2016) can be generated by solar panels over parking lots. Level 1 charging is 110 volt charging with about 2 to 3 kW of power flowing into the EV. This value is about the same as the power produced by solar panels over one parking place [2]. For workplace charging, level 1 charging can provide 16-30 kWh of electricity during a working day, which is sufficient to travel 48–90 miles (77–145 km). Most workers travel less distance than this to come to work. Level 1 EVSE is often supplied with the EV at the time of purchase, so an electrical outlet with power and a 30 amp fuse is what is needed as part of the charging infrastructure in the parking lot. In the National Academies report [25], workplace charging is given the highest priority after home charging. For those EVs with limited range, workplace charging may allow use of an EV with a range of 120 km to be used to travel to work where the distance to work is 70 km.

The solar panels can be connected to the local electrical grid such that power can flow into the grid and the grid can provide power for EV charging. Most parking lots with solar panels can have a mix of level 1 and level 2 EVSE. Level 2 charging delivers about 6 kW of power with a 240 volt power supply. The largest battery packs in EVs are about 70 kWh, which means it takes about 10 hours with level 2 charging to add 60 kWh to an EV with a large battery storage capacity. The EVSE for level 2 is normally fixed to the electrical power supply and is part of the charging station. The cost of EVSE for level 2 ranges from about $1200 to $4000; some EVSE includes both level 1 and level 2 charging [2].

In Kansas, 16 kWh per parking place per day is a good estimate for the electricity generated by solar panels. There are of the order of 800 million parking spaces in the U.S., but not all of them are positioned appropriately for addition of solar panels. With about 200 million parking spaces with solar panels, about 3 billion kWh/day can be generated, which is about equal to the daily electricity requirement to allow most drivers to drive about 13,000 miles/year (21,000 km/year). The annual production of electricity from covering 200 million parking spaces with solar panels is about equal to 1/4 of the electricity production in the USA [2].

The cost of installation of solar powered charging stations varies with location and the local economy; values from $10,000 to 20,000 per parking space have been used in [2]. When an entire parking lot is covered with solar panels, the installation costs are less than for a small project with several parking spaces. Generally, the operating cost for an EV will be less than that for a gasoline vehicle. The value of shaded parking contributes positively to the economics of solar powered charging stations.

Tesla has introduced fast charging with solar panels to generate electricity and battery storage to avoid high demand charges from electric utilities [2]. Fast charging is more expensive to install and the cost of the electricity may be more because of demand charges if grid power is used. Public fast charging EVSE is more expensive because of the rate of power flow and the need to work with all customers. Costs as high as $122,000 per charge station have been reported [25] for the West Coast Electric Highway project that is designed to provide fast charging along I-5 in California, Oregon, and Washington. It may be necessary to charge about $0.20 to $0.25 /kWh for electricity at fast charging stations in order to pay for the capital and operating costs [25]. The Sacramento Municipal Utility District charges $0.22 /kWh for both level 2 and DC fast charging in their solar parking lot (Personal communication with R. Troute, August 12, 2016). Tesla has provided free charging at its supercharger stations to Tesla owners. With the 200 mile range and supercharger stations, it is possible to travel to most places in the United States in a Tesla EV.

One of the developments needed is to standardize the connection to the vehicle for fast charging. Presently there are three different designs that are commonly used: 1. Combined Charging System (CCS) which includes the J1772 Level two charging port and an added port to allow for fast charging; 2. CHAdeMO quick charge standard developed by Toyota, Nissan and Mitsubishi; and Tesla Supercharger. Tesla has more than 650 locations with Superchargers in the world. There are more than 1500 CHAdeMo level 3 chargers in the U.S. Some sites have both CHAdeMo and CCS equipment available.

One of the barriers associated with the deployment of plug-in vehicles is range anxiety because the number of locations to charge EV batteries is limited. The addition of solar powered charging infrastructure has the benefits of reducing range anxiety greening the electrical grid, and providing shaded parking. In Arizona, there are many locations with solar panels and shaded parking where there are no EV charging stations because they were installed before EVs were popular and shade is very beneficial on hot days.

## 8. Electric Buses

Significant progress has been made with respect to the electrification of bus transportation [2],[29]–[35]. Chicago Transit Authority (CTA) purchased two electric buses in 2014 that were made by New Flyer Industries, and has been testing them in daily revenue service since October 2014 [30]. Each bus has a 300 kWh battery pack which is sufficient for a range of 80 miles. The city plans to add 20 to 30 additional electric buses over the next few years [30]. The CTA estimates that each bus will save $25,000 in fuel and reduce public health costs by $55,000 per year because of better air quality.

Proterra is a new company, founded in 2004, that has a goal of electrifying bus transportation with Proterra buses [29],[31],[35]. It was the first company to deliver a full-size electric bus that met California's Zero Emissions Bus Rules [31]. Eudy et al [35] have reported on the results of an evaluation of the technology of 12 Proterra electric buses in service in California. The energy required was 2.15 kWh/mile (1.34 kWh/km). While the electric motor does not use power when the bus is stopped to pick up passengers, the lighting and air conditioning use power. Maintenance costs were $0.16 /mile ($0.10 /km). Proterra buses are available with different amounts of battery storage up to about 330 kWh. With larger battery packs it is possible to avoid charging during peak power demand times and make greater use of off-peak times to charge the buses. Proterra is a U.S. company with manufacturing in South Carolina.

James Ayre [32] has reported on electric bus service in Gothenburg, Sweden, where Volvo electric buses have been evaluated after a year of use on Route 55. The report indicates that the reduced noise level and better air quality were important qualities. Those who used the buses also liked the availability of free Wifi [32].

The Chinese company BYD (Build Your Dreams) is building electric buses for sale in China, the U.S. and Europe [33],[34]. The company has a manufacturing plant in Lancaster, California. It hopes to produce and sell 300 electric buses at this plant in 2016. It is also producing and selling electric buses in China. The new buses sold to France will be used to improve air quality in Paris.

The electric buses are becoming popular with cities because they have lower operating costs and they improve air quality. Many cities have started to order electric buses. There is the hope that this transformation to electric buses will happen rapidly and that one decade from now more than half of the buses will be electric buses. This will help reduce greenhouse gas emissions and improve air quality.

Four electric bus manufacturers, BYD, New Flyer Industries, Proterra, and Volvo have been mentioned above. There are a sufficient number of companies to have some competition, which helps to encourage product quality and keep prices down.

## 9. Conclusions

Air in many locations in the world has concentrations of particulates, volatile organic compounds, and nitrogen and sulfur oxides from combustion processes that affect human health. If all combustion processes associated with the generation of electricity and transportation were replaced by sustainable energy generation and electric vehicles, air quality would improve significantly.

Solar powered charging infrastructure has the potential to increase the availability of charging stations and reduce range anxiety. Costs of solar panels, wind energy, and batteries are decreasing, and this has resulted in increased sales of EVs, and greater numbers of installations of wind farms and solar panels. In 2015, wind power was ranked number 1 and solar power was ranked number 2 for reported new electric generating capacity. The large batteries in EVs can be charged with lower cost electricity when time of use prices is available. The nexus of solar powered charging infrastructure, electric vehicles, and renewable energy has the potential to improve public health by improving air quality and reducing carbon emissions.

The very large cities in the world would have significant social, environmental, and economic benefits from transitioning to plug-in electric vehicles and renewable energy. Electric buses powered by electricity from wind and solar installations are economical because of the health benefits associated with better air quality.

The great health benefits associated with electric vehicles and electric power from renewable sources that have been achieved in California and Norway are available to be duplicated in many other locations in the world. Since prices of electricity from wind and solar, batteries, and electric vehicles are going down, there are many good reasons to move forward with sustainable energy initiatives which will reduce greenhouse gas emissions and improve air quality and health.
